# Phenotypic prevalence of extended spectrum beta-lactamases among enterobacteriaceae isolated at Mulago National Referral Hospital: Uganda

**DOI:** 10.1186/s13104-017-2786-3

**Published:** 2017-09-06

**Authors:** Lucas Ampaire, Emmanuel Nduhura, Izale Wewedru

**Affiliations:** 1 Department of Medical Laboratory Sciences, Mbarara University of Science and Technology, P.O Box. 1410, Mbarara, Uganda; 2 Microbiology Department, Mulago National Referral Hospital, Kampala, Uganda

**Keywords:** ESBLs prevalence Mulago, Mulago ESBL prevalence, Phenotypic ESBLs prevalence

## Abstract

**Objective:**

*Enterobacteriaceae,* common causes of health care associated and community acquired infections are mainly treated with beta-lactam agents. Our study objective was to determine the prevalence and common enterobacteriaceae pathogen producing extended spectrum beta lactamases (ESBLs). The isolates were recovered from various clinical specimens. This was cross sectional study conducted between July 2016 and September 2016 at Mulago National Referral Hospital, Uganda. We used ChromID™ ESBL agar (Biomerieux SA, Lyon, France) and Vitek2 compact system GN83 card (BioMerieux Inc, Hazelwood, Missouri, USA) to detect and confirm presence of phenotypic extended spectrum beta lactamases producing pathogens respectively.

**Results:**

Of the 261 tested clinical isolates, 35 (13.4%) were identified as ESBLs producing bacteria. *Escherichia coli* predominated in the samples [18 (51.4%)], presenting the highest frequency of ESBLs producing, followed by *Klebsiella pneumonia* [10 (28.5%)], *Proteus mirabilis* [4 (11.4%)], *Enterobacter* sp. [2 (5.7%)] and least among *Acinetobacter baumanii* [1 (2.8%)].

## Introduction

*Enterobacteriaceae* are common causes of hospital and community acquired infections. The key challenge in their treatment has been their tendency to develop resistance to commonly used beta-lactam agents. Yet, these drugs are the main stay of treatment especially in low developed countries [1].

Limited therapeutic options for extended spectrum beta-lactamase (ESBL)-producing bacteria result in poor prognosis, high carrier rate, infectivity and an increased capacity to cause hospital acquired infections [2]. Many studies report *Klebsiella pneumonia*e and *Escherichia coli* as the most common ESBL producing bacterial species. However, other bacterial species in the families of Enterobacteriaceae and Pseudomonadaceae are also known to produce such enzymes [4–8].

The ESBL prevalence varies across Africa, being highest in North Africa (16.4–77.8%), and least in South Africa (8.8–13.1%). In East Africa, studies report a prevalence ranging from 37.4 to 62.8% [3, 4]. The wide regional differences underscore the need to take into account at the level of the country, the region, the hospital, and at times the individual hospital unit when making decisions about empirical therapy for serious infections.

The prevalence of ESBLs-producing bacteria is unknown in Mulago hospital that serves as a National Referral hospital for Uganda. The study used chromogenic agar and Vitek compact system GN83 (an integrated automated system) for the detection of ESBLs in addition to routine bacterial identification and susceptibility testing. These methods were considered because of their affordability in resource constrained settings and their usefulness in rapid detection of ESBL producers compared to more expensive genotypic detection techniques [9].

## Main text

### Methods

#### Study design

This was a cross sectional study. Enterobacteriaceae isolates obtained from various clinical specimens submitted from various hospital units within Mulago National Referral Hospital were tested for phenotypic expression of ESBLs. The hospital units included medical assessment center, surgical units and private outpatient department.

#### Study area

The study was conducted at Mulago National Referral Hospital, located about 3 km from Kampala city centre, Kawempe division. It serves as both Makerere University Teaching Hospital and then main National Referral Hospital for Uganda. Clinical specimens were analysed at Microbiology Laboratory of Mulago Hospital.

#### Sample size estimation

We calculated a sample size of 261 using Kish and Leslie, 1965 formula. This was based on an estimated 17% ESBLs prevalence, 80% power with a 95% confidence and precision of ∼5%.

#### Sample collection, processing and data analysis

All clinical specimens for culture and sensitivity testing were collected and transported in accordance to the locally approved standard operating procedures. These samples were then directly cultured on the conventional media including MacConkey agar (BDH), 5% sheep blood agar, and chocolate agar. The inoculated MacConkey agar (BDH) and 5% sheep blood agar were incubated aerobically at 35–37 °C for 18–24 h, while chocolate agar was incubated in anaerobic condition with similar temperature and time range. The bacterial growth was noted based on colonial characteristics and identified by Gram staining, triple sugar iron agar (TSIA) colonial characteristics, and analytical profile index (API20E).

On isolation as member of enterobacteriaceae, a microorganism suspension (equivalent to 0.5 McFarland) was prepared in sterile saline and then sub-cultured onto ChromID™ ESBL agar (Biomerieux SA, Lyon;France) for screening for ESBL production. ESBL production was interpreted by colonial color differences as described by Manufacturer (*Escherichia coli* was identified by pink color production, *Klebsiella, Enterobacter, Serratia, and Citrobacter* (KESC) by production of either green, brownish-green or blue colour while *Proteus, Providencia, and Morganella* by production of dark brown to light brown colour).

For confirmation of ESBL production, Vitek2 compact system GN83 card (bioMerieux Vitek, Hazelwood, Missouri, USA) was inoculated with standard microorganism suspensions using an integrated vacuum apparatus. The GN83 susceptibility card contained Amikacin, Amoxicillin/Clavulanic acid, Ampicillin, Ampicillin/Sulbactam, Aztreonam, Cefazolin, Cefepime, Cefotaxime, Cefoxitin, Ceftazidime, Ceftriaxone, Cefuroxime, Cefazolin, Ciprofloxacin, Gentamicin, Meropenem, Nitrofurantoin, Piperacillin/Tazobactam and Trimethoprim/Sulfamethoxazole.

A test tube containing the microorganism suspension was placed into the GN83 cassette and the identification card placed in the neighboring slot while transfer tube was inserted into the corresponding suspension tube. After the vacuum was applied and air re-introduced into the station, the microorganism suspension was forced through the transfer tube into micro-channels that filled all the test wells (followed manufacturer’s instructions). Interpretation was made after 24 h incubation.

Following manufacturer’s instructions, expression of an ESBL was made by comparison of Logarithmic reduction in growth within the well(s) containing clavulanic acid and or cefotaxime/ceftazidime in combination to the well not containing clavulanic acid. *Klebsiella pneumoniae* ATCC 700603 and *Escherichia coli* ATCC 25922 procured from the Uganda National Health Laboratories Services (UNHLS) were used as control organisms in this study.

All data was analyzed using STATA software version 12.1 (StataCorp, College Station, TX, USA) to generate descriptive statistics which included proportions, frequencies and percentages that were presented in form of tables.

### Results

A total of 2814 clinical specimens were analysed, out of which 261 non repetitive isolates identified as enterobacteriaceae were tested for ESBL production (Table 1). Out of the 261 isolates, 146 (55.9%) were identified as *Escherichia coli*, 68 (26.1%) as *Klebsiella pneumonia*e, 14 (5.4%) as *Proteus mirabilis*, 8 (3.1%) *Serratia sp*, 7 (2.7%) as *Salmonella typhi*, 6 (2.3%) as *Acinetobacter baumanii*, 5 (1.9%) as *Morganella morganii,* 4 (1.5%), *Enterobacter* sp. and the rest as *Providencia* 3 (1.1%) (Table 2).

**Table 1:** Sources of clinical specimens and ESBL distribution

Hospital unit	No. of samples	ESBLs(n)
Medical assessment center (MAC)	1843	19
Surgical units	551	12
Private out patient	420	4

**Table 2:** Distribution of isolates obtained in the study

Isolate name	n (%)
*Escherichia coli*	146 (55.9)
*Klebsiella pneumoniae*	68 (26.1)
*Proteus mirabilis*	14 (5.4)
*Serratia* sp.	8 (3.1)
*Salmonella typhi*	7 (2.7)
*Acinetobacter baumanii*	6 (2.3)
*Morganella morganii*	5 (1.9)
*Enterobacter* sp.	4 (1.5)
*Providencia* sp.	3 (1.1)

On screening for potential ESBL production using chromID™ agar, Seventy-four (74) isolates were suggestive of which; *E. coli* (41,55.4%) were the majority followed by *K. pneumoniae* (19,25.7%) and the least as *A. baumanii* (3,4.0%). On confirmation with Vitek2 Compact AST GN 83 card only 35 (13.4%) isolates out of the 74 were confirmed as phenotypic ESBL producers. ESBL production was more common among *K. pneumoniae* (28.6) and *E. coli* (51.4) compared to other isolates (Table 3).

**Table 3:** Prevalence of phenotypic ESBLs producing isolates

Isolate	ESBL production n (%)	
	Chrom agar	Vitek
*E. coli*	41 (55.4)	18 (51.4)
*K. pneumoniae*	19 (25.7)	10 (28.6)
*Proteus mirabilis*	7 (9.5)	4 (11.4)
*Enterobacter* sp.	4 (5.4)	2 (5.7)
*A. baumanii*	3 (4.0)	1 (2.9)
Total	74	35

### Discussion

In this cross sectional study, we found moderate prevalence of Phenotypic ESBL production (13.4%). This is however, lower compared to that recorded in Kabale Regional Referral Hospital, Uganda (89%) [4], Mbarara Regional referral Hospital, Uganda [7], Khartoum Teaching Hospital, Sudan (45.1%) [10], Kenya and Ethiopia (62.8%) [3], India (52.49%) [11] but closely related to that recorded in Lebanon (15.4%) [12] and Tunisia (11.7–77.8%) [3]. This may be attributed to difference in methods used for phenotypic detection of ESBL production. In our present study, automated Vitek compact system AST84 was used compared to the manual combined disk diffusion technique (whose sensitivity and specificity are low) that was used in many of the studies is reported before. These findings contribute data necessary to prevent, control and contain effectively the antibiotic-resistant pathogens such as ESBL producers in resource-limited settings.

In our present study *E. coli* and *K. pneumoniae* were the commonest ESBL producers and most common isolated among the enterobacteriaceae. Alarmingly, these bacterial pathogens are commonly associated with nosocomial infections. Our findings are similar to findings from other studies; in Kabale 44 and 52% [4], Mbarara, 59.5% [7], India 67.04% [11]. But this contrasts other studies in which *Enterobacter* sp. and *Acinetobacte*r have been indicated as most prevalent ESBLs-producer in clinical isolates [13].

The prevalence of ESBL-producing *E. coli* and *Klebsiella* sp. detected in this study is of great concern at both Mulago Hospital and other healthcare settings in Uganda, which requires sound and committed sustainable infection control measures including antimicrobial management and routine detection of ESBL-producing isolates. Future research on ESBL in Uganda should focus on elucidating risk factors for ESBL carriage and infection, particularly among surgical patients and Out patients.

### Conclusion

Knowing local ESBL carriage prevalence can help guide clinical care. Phenotypic screening using automated systems improves the sensitivity and specificity, turnaround time and could be the cheapest strategy to routinely use for screening and confirming ESBL producers.

## Limitations

- The study was unable to determine whether the infectious agents were hospital acquired or Community acquired.
- Due to limited funds we were unable to do molecular detection for the genes that may not have been expressed.
- Being a cross-sectional study, the study was unable to determine risk estimates and or follow-up of colonized individuals to be able to determine whether these were carriers.

## Abbreviations

ESBLs: extended spectrum beta lactamases
TSIA: triple sugar iron agar
